# Anti-Inflammatory and Proliferative Properties of Luteolin-7-O-Glucoside

**DOI:** 10.3390/ijms22031321

**Published:** 2021-01-28

**Authors:** Alessandro De Stefano, Sabrina Caporali, Nicola Di Daniele, Valentina Rovella, Carmine Cardillo, Francesca Schinzari, Marilena Minieri, Massimo Pieri, Eleonora Candi, Sergio Bernardini, Manfredi Tesauro, Alessandro Terrinoni

**Affiliations:** 1 Centre of Space Biomedicine, Department of Systems Medicine, University of Rome Tor Vergata, 00133 Rome, Italy; alessandrodestefano6@virgilio.it (A.D.S.); didaniele@med.uniroma2.it (N.D.D.); valerovix@yahoo.it (V.R.); mtesauro@tiscali.it (M.T.); 2 Department of Industrial Engineering, University of Rome Tor Vergata, 00133 Rome, Italy; sabrina.caporali93@gmail.com; 3 Department of Clinical Sciences and Translational Medicine, Cattolica University of Rome, Via Montpellier, 1, 00133 Rome, Italy; carmine.cardillo@unicatt.it; 4 Internal Medicine, Policlinico A. Gemelli Istituto di Ricovero e Cura a Carattere Scientifico, 00133 Rome, Italy; francescaschinzari@gmail.com; 5 Department of Experimental Medicine, University of Rome Tor Vergata, Via Montpellier, 1, 00133 Rome, Italy; marilenami@gmail.com (M.M.); massimo.pieri@uniroma2.it (M.P.); candi@uniroma2.it (E.C.); bernardini@med.uniroma2.it (S.B.); 6 Laboratory of Biochemistry, IDI-IRCCS Fondazione Luigi Maria Monti, Via Monti di Creta 104, 00167 Rome, Italy

**Keywords:** flavonoids, luteolin-7-O-glucoside, hydroxylation, inflammation, STAT3

## Abstract

Flavonoids display a broad range of structures and are responsible for the major organoleptic characteristics of plant-derived foods and beverages. Recent data showed their activity, and in particular of luteolin-7-O-glucoside (LUT-7G), in reduction of oxidative stress and inflammatory mechanisms in different physiological systems. In this paper, we tried to elucidate how LUT-7G could exert both antioxidant and anti-inflammatory effects in endothelial cells cultured in vitro. Here, we showed that LUT-7G is able to inhibit the STAT3 pathway, to have an antiproliferative action, and an important antioxidant property in HUVEC cells. These properties are exerted by the flavone in endothelial through the transcriptional repression of a number of inflammatory cytokines and their receptors, and by the inhibition of ROS generation. ROS and STAT3 activation has been correlated with the production of oxysterols and other hydroxylated fatty acids, and they have been recognized important as players of atherogenesis and cardiocirculatory system diseases. The analysis of the general production pathway of these hydroxylated species, showed a strong decrease of cholesterol hydroxylated species such as 7-alpha-hydroxicholesterol, 7-beta-hydroxicholesterol by the treatment with LUT-7G. This confirms the anti-inflammatory properties of LUT-7G also in the endothelial district, showing for the first time the molecular pathway that verify previous postulated cardiovascular benefits of this flavone.

## 1. Introduction

An increasing number of plant-derived molecules, such as flavonoids, which are a class of plant secondary metabolites have shown positive effects on several conditions such as cardiovascular and immunological diseases and in cancer [1,2]. They display a large range of structures and, they are responsible for the major organoleptic characteristics of plant-derived foods and beverages, particularly color and taste properties and they contribute to the nutritional qualities of fruits and vegetables [3]. Recent developments in the understanding of oxidative stress and inflammatory mechanisms of the cardiovascular system tried to elucidate how flavonoids exert beneficial cardiovascular effects because of their anti-oxidant and anti-inflammatory properties, coupled with their capacity to modulate key cellular function [4,5]. 

As reported, a diet with an adequate consumption of fruits and vegetables have been long established as primary prevention of cardiovascular disease (CVD). Vascular inflammation and lipid accumulation in the arterial wall are hallmarks of atherosclerosis, which represent the prominent risk factor in the onset of cardiovascular disease (CVD). Atherosclerosis is a chronic inflammatory process affecting the arteries walls. It is well noted during the vascular inflammation process that proinflammatory cytokines such as IL-6 are released from endothelial cells (EC) and vascular smooth muscle cells (VSMC) contributing to vascular damage. Peculiarly, IL-6 stimulation of the Janus kinase (JAK)/STAT3 pathway is a key mechanism that regulates EC inflammation, SMC migration, and proliferation. The gp130 phosphorylation by JAK induces phosphorylation and dimerization of STAT3, which translocating to the nucleus activates the expression of proinflammatory genes [6].

Yet, as stated, the accumulation of lipids in the arterial wall can trigger the development of atherosclerosis. Certainly, linoleic free fatty acids and hydroxy acids (such as 13-HODE) induced atherosclerotic lesions by inducing a vascular inflammation cascade. The oxidized linoleic acid contained in low-density-lipoprotein (LDL) leads to harmful oxidized linoleic acid metabolites (OXLAMs) formation with the consequent activation of NF-κB and the increasing of proinflammatory cytokines, endothelial adhesion molecules, as well as chemokines [7]. Atherosclerosis is also induced when the vascular dysfunction in endothelium occurs following the nitric oxide (NO) impairment consequent to inflammation [8]. 

**Figure 1:**
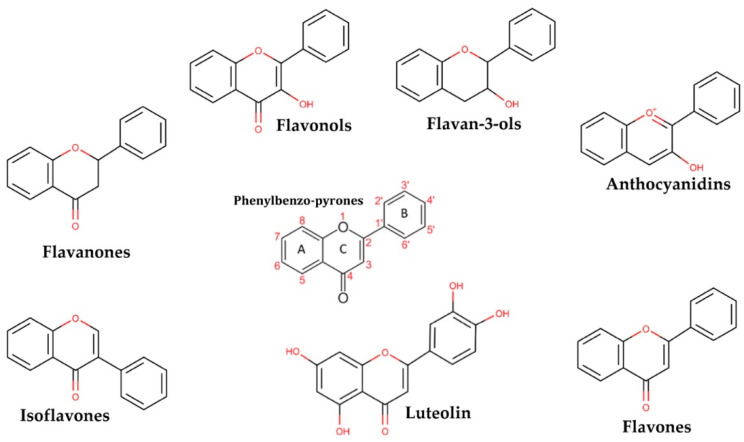
The picture shows the derivatives of phenylbenzo-pyrone representing the basic structure of a wide class of molecules.

The glycosylated form of luteolin (3′,4′,5,7-tetrahydroxyflavone, LUT-7G) is one of the most widespread flavones with anti-oxidant, anti-tumour, anti-inflammatory, and anti-apoptotic activities [9,10]. Belonging to the flavone group of the flavonoids, it shows a C6-C3-C6 structure holding two benzene rings (A,B), and a third, oxygen-containing (C) ring, with a 2−3 carbon double bond. It is retrieved in a glycosylated form in plants, with the glycoside probably hydrolyzed during absorption, even if the definitive mechanism has not been completely elucidated. Luteolin is dehydroxylated in positions 3’ and 4’ (Figure 1).

The hydroxyl moieties and 2−3 double bond are important structural features in this compound and are associated with their biochemical and biological activities, like the antioxidant activity. It has been proven that this natural compound may elicit strong cardiovascular protective activities via complex signal transduction pathways and target effectors and by vasodilation mechanisms [11]. Notably, it has been demonstrated in keratinocytes that LUT-7G is able to counteract the gene transcription exerted by IL-22 and IL-6, by the activation of the JAK/STAT3 pathway and consequently proinflammatory gene transcription. It has been demonstrated that the inhibition of this pathway by LUT-7G is obtained by the impairment of the nuclear translocation of STAT3 [12]. Moreover, LUT-7G has been shown to have a strong antioxidant effect in growing cells and also has important effects on cellular energy production [13]. Suppression of proinflammatory cytokine responses via targeting the JAK/STAT3 pathway and the possible reduction of lipid accumulation and oxidation could be useful as a potential therapeutic strategy in patients with a high cardiovascular risk [6]. For this purpose, we analyzed the anti-proliferative and anti-inflammatory effects of luteolin in human umbilical vein endothelial cells (HUVEC). In particular, the aim of our study was to investigate and demonstrate in the HUVEC system, the effect of LUT-7G on the STAT3 pathway previously described in keratinocytes. Based on the fact that the LUT-7G/STAT3 pathway turns out to be conserved in both cell lines, we speculate that the flavone could produce cardiovascular benefits, through the demonstrated inhibition of STAT3, the downregulation of the target gene involved in the inflammation, and by inhibiting ROS production in endothelial cells. ROS have been demonstrated to be able to generate high oxysterol levels, like 7- ketocholesterol, and 7α-hydroxycholesterol in endothelial cells. These chemical species have been correlated with vascular aging and atherosclerosis [14,15,16]. For this reason, we extracted data on the modification of lipid profile and peroxidation obtained from human epidermal keratinocytes (HEKn) treated with LUT7-G, to investigate the general pattern of modulation of the different species of hydroxylated lipids. 

## 2. Results

### 2.1. The LUT-7G Treatment Shows Inhibition of STAT3 and Reduction of Endothelial Cell Proliferation

**Figure 2:**
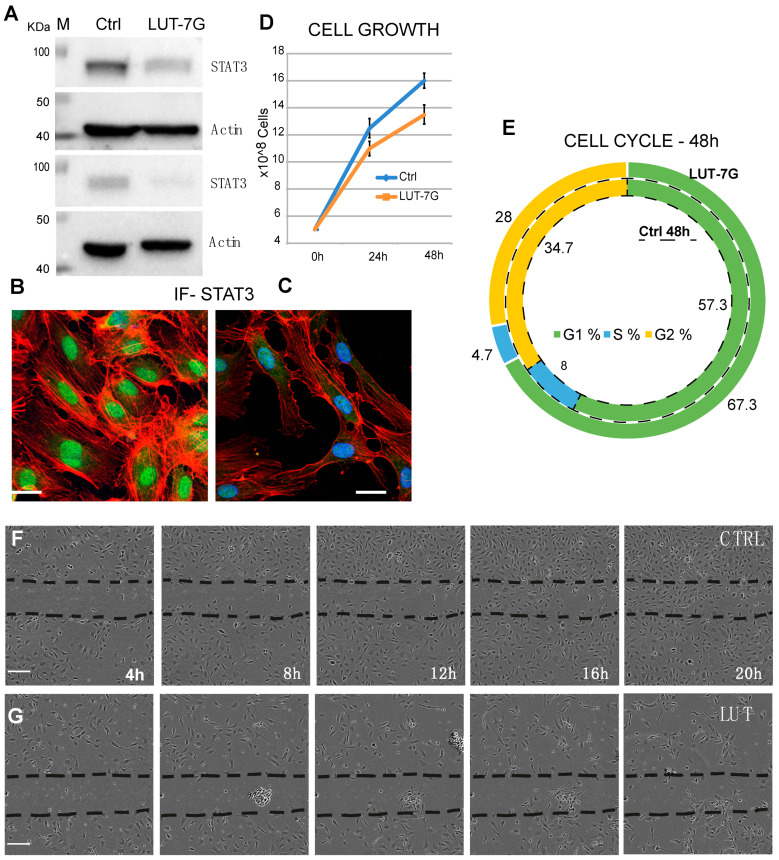
LUT-7G treatment of endothelial cells. (**A**) WB showing the regulation of STAT3 protein before (left lanes) and after LUT-7G treatment (right lanes, two independent experiments). (**B**,**C**) Immunofluorescence analysis showing STAT3 in the nucleus (active STAT3) in untreated cells and STAT3 nuclear depletion after 48 h of LUT-7G treatment (bars = 15 μm). (**D**,**E**) Cell Growth and Cell cycle analysis showing proliferation reduction and G1 enrichment in treated samples. (**F**) Scratch assay in untreated cells, (**G**) same assay from 4 to 20 h (bars = 60 μm).

As previously demonstrated, LUT-7G is able to regulate the cell cycle, differentiation, and inflammatory pathway in keratinocytes via STAT3 inhibition [12]. The activation of the JAK/STAT3 pathway is a key mechanism regulating endothelial inflammation [17], vascular smooth muscle cell migration, and proliferation. Moreover, it has been shown that this pathway is involved in the promotion of the development of cardiovascular diseases such as atherosclerosis [18]. To investigate the pathway in the correct environment, we performed experiments using endothelial cells (HUVEC). These had been treated with LUT-7G to investigate its effects on the STAT3 pathway. Interestingly, we found that LUT-7G is able to reduce the STAT3 production (Figure 2A). Moreover, confocal fluorescence analysis demonstrates a depletion of STAT3 in treated cells (Figure 2B,C). These results support the hypothesis that also in this cellular type, LUT-7G counteracts the pro-proliferative, and pro-inflammatory action of STAT3. A probable mechanism of action of LUT-7G on STAT3 transcription factor could be due to the physical interaction between the flavone and the transcription factor [19]. Specifically, this has been evidentiated, employing a molecular modeling showing a possible interaction between STAT3 SH2 domain and luteolin [19]. We performed a similar analysis using the glycosylated form of the flavone and the STAT3 mouse X-ray crystal structure (same of [19]). The molecular docking analysis detects a binding energy of over −8.0 kcal/mol for the first three clustered solutions in crevices on the STAT3 surface and of over −7.0 kcal/mol for the remaining seven solutions. In addition, this computational modeling shows that LUT-7G could bind to the SH2 domain of STAT3 (Figure S1), thus confirming that LUT-7G may block the activity of STAT3 and could be considered as candidate as inhibitor of this transcription factor. 

To further assess this action, we performed experiments analyzing the cell grow and cell cycle. As shown in panel D of Figure 2, the total number of cells after 48 h is lower in the sample treated with LUT-7G. The flavone has no effects on viability of HUVEC cells, since apoptosis is similar in the two samples (9% vs. 9.5%), but they reduce their replication time. The cycle analysis shows an important diminishment of the S phase, with an increase of the G1 phase, leading to consider this as a G1 block (Figure 2E). This phenomenon is generally due to the induction of a specific differentiation program, and a similar result has been obtained in the treatment of keratinocytes with the flavone. The cytostatic effect of the molecule is clearly visible in a scratch assay experiment, in which the cells have been seeded and after 24 h a scratch has been created in the culture plate. The closure of the scratch has been analyzed starting from 4 up to 20 h (Figure 2F,G). The results show that the scratch is profoundly delayed by the treatment with LUT-7G (Figure 2G) with respect to the control (Figure 2F) in which a complete closure of the scratch is already visible in the 16 h time point. This regulation on cell grow could be also the consequence of another effect of LUT-7G, since it is known that LUT-7G is able to block also glucose metabolism [13].

**Figure 3:**
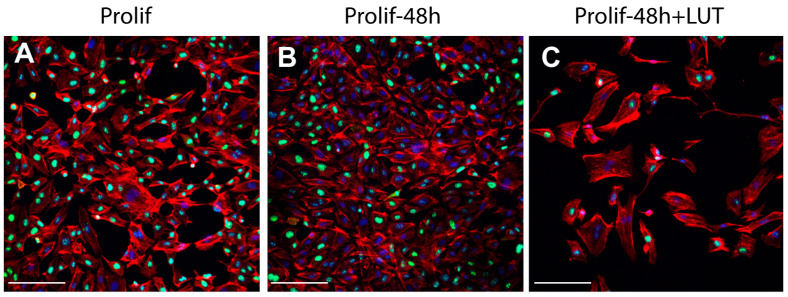

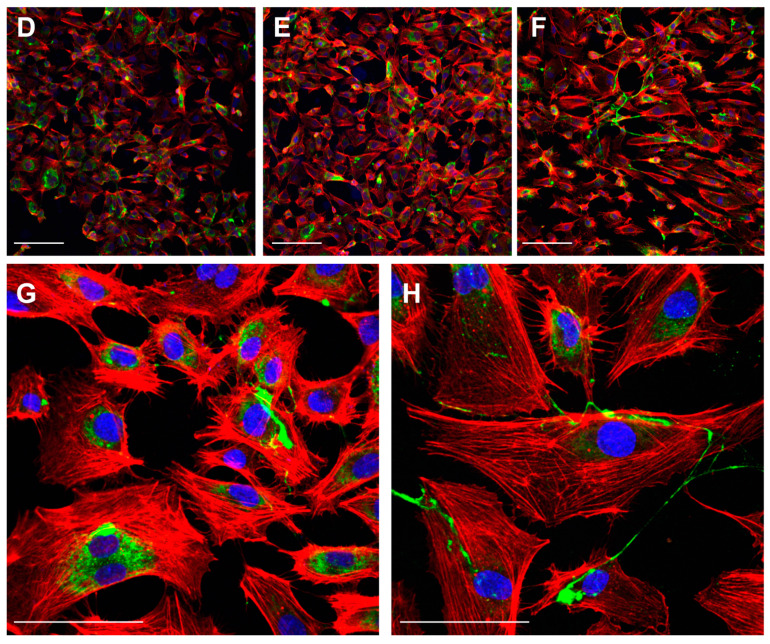
LUT-7G treatment in endothelial cells. (**A**) immunofluorescence analysis with Ki67 staining (green) of HUVEC cells in proliferating condition. (**B**) After 48 h without treatment. (**C**) After 48 h and LUT-7G treatment. (**D**–**F**) Same time course and treatment with vWF staining (green) (bars = 60 μm). (**G**,**H**) Higher enlargement of vWF staining, showing a cytoplasmic location in untreated cells (**G**) and a membrane staining in treated cells. Indeed, treated cells show an important reduction of vWF synthesis. In the picture, phalloidin is stained in red, and nuclei in blue (DAPI (4′,6-diamidino-2-phenylindole); bars = 60 μm; Prolif = proliferating endothelial cells).

Interestingly, the analysis of proliferation markers such as Ki67 demonstrated the reduction of the proliferation potential of endothelial cells after the treatment with LUT-7G. As it is clearly visible (Figure 3), the treatment with this molecule is able to reduce both the total number of cells and the positivity to Ki67 at a nuclear level. In fact, in absence of treatment, the cells continue to proliferate, increasing their number (Figure 3B vs. Figure 3A), while in the presence of LUT-7G, the number is reduced and the signal at the nuclear level is markedly decreased (Figure 3C vs. Figure 3A). Endothelial cells are also responsible for the synthesis of the von Willebrand factor (vWF), a glycoprotein synthesized in the endoplasmic reticulum of these cells, which is important in the coagulation cascade and vascular inflammation [20,21,22]. The vWF undergoes modifications such as cleavage and multimerization processes in the Golgi apparatus [23], and plays a role in different pathways of the angiogenesis. Importantly, the re-localization of vWF at the cell surface, and its interaction with αvβ3 integrin stabilizes its expression and suppresses VEGFR-2 function, blocking downstream signaling necessary for angiogenesis [24,25], and importantly endothelial cell proliferation [26]. The staining with anti vWF antibody of HUVEC cells effectively show this protein to be mainly localized in the cytoplasm, while in those treated with LUT-7G, the factor seems to be localized on the cell surface (Figure 3D–F). Higher enlargement finely shows a membrane location of vWF in LUT-7G treated cells, compared to the untreated cells (Figure 3G) in which the localization is cytoplasmic. Moreover, an evident decrease of vWF in cytoplasmic location is also evident (Figure 3H) in treated cells, thus confirming a pro-differentiating and antiproliferative effect of LUT-7G on endothelial cells. 

### 2.2. Effects of LUT-7G Treatment in Modulating Inflammatory Citokines Genes

To investigate the possible anti-inflammatory role of the flavone in endothelial cells, we used cultured HUVEC, treated with a concentration of 20 µM of LUT-7G for 48 h and then extracted the RNA. A number of genes of the inflammatory pathway have been analyzed by RTqPCR (see Methods) the main results (+/−2-fold changes, and *p* Value < 0.05) have been reported in Table 1, the complete table in Table S1. As is visible in the tables (Table 1 and Table S1), most genes are downregulated and a restricted part of them are upregulated. Using literature data, we identified in regulated genes those involved in vascular inflammation and atherosclerosis. As reported, the treatment reduces the transcription of a number of cytokines and their receptors, with inflammatory properties. Among them, CCL1, a chemokine secreted by activated T cells with chemotactic activity for monocytes, which is involved in immunoregulatory and inflammatory processes. Notably, CCL1 binds to the chemokine (C-C motif) receptor 8 (CCR8), a transmembrane protein, important for the migration of various cell types into inflammatory sites, that also results downregulated. Importantly, the presence of the CCR8 antigen on the luminal endothelial surface of human atherosclerotic plaques has been demonstrated [27]. Another is CCL11, which is a secreted chemokine with chemotactic activity for eosinophils. This chemokine binds to CCR3, that also results highly downregulated with treatment. Another example of a downregulated cytokine is CCL3, which also plays a role in inflammatory response stimulating macrophages. Moreover, CXCL12 results highly downregulated, since it is responsible for chemoattractant activity on T-lymphocytes and monocytes, and is involved in immune surveillance, inflammation response, in surveillance of tumour growth and metastasis [28,29,30]. Another downregulated gene is LTB4R, a class of receptors activated by the endogenous ligands leukotriene (LT), lipid mediators synthesized from arachidonic acid by lipoxygenase enzymes, important in inflammatory signaling pathways. Another repressed transcript is IL-1β, which is a member of the interleukin 1 family of cytokines. This cytokine is an important mediator of the inflammatory response. Recently, studies evaluated clinically applicable interventions that interfere with IL-1 action in atherosclerosis therapy [31]. Among regulated genes, the CCR7 receptor and its ligands (CCL19-CCL21) result in be highly downregulated. These also play a role in the atherosclerosis by recruiting T-cells and macrophages in the atherosclerotic plaques, enhancing inflammatory responses [32,33]. In Table 1, we tried to summarize the concomitant regulation of chemokines and their related receptors. In our study, we found downregulated IL-36A (IL1F6), a member of the IL-1 family. This gene is important since it is involved in vascular inflammation by activating proinflammatory cytokines such as TNF-alpha [34], IL-8, and IL-6. [35]. Its downregulation by LUT-7G treatment could confirm the role of the flavone in the mitigation of vascular inflammation. Specifically, IL-6 promotes the activation of JAK/STAT3 and PI3/AKT inflammatory signals in endothelial cells [36]. These lead to endothelial dysfunction and the development of atherosclerosis [37]. In our gene array analysis on endothelial cells, we did identify upregulated genes such as IL-10RB which mediate the immunosuppressive signal of interleukin 10, exerting an anti-inflammatory effect. Moreover, ICEBERG caspase-1 inhibitor is also found to be upregulated since its pivotal role in counteracting the generation of IL-1beta [38].

### 2.3. LUT-7G Treatment Effects Reverts IL-22 Promoted Inflammatory Genes

Since a direct link has been demonstrated between inflammatory diseases of the skin, associated with chronic inflammation such as psoriasis, with atherosclerosis, and of cardiovascular diseases [39,40,41], we also used a keratinocyte model, in which a psoriatic phenotype has been induced with IL-22 treatment [12,13]. Modulated genes have been analyzed using a wider array model (Biorad PCR panel, see Methods). The panel contains 189 human genes, involved in the pathogenesis of psoriasis. It contains genes important for keratinocyte differentiation and proliferation as well as mediators. mRNAs from treated keratinocytes (with IL-22 alone or in combination with the flavone) were used for the purpose. 

**Figure 4:**
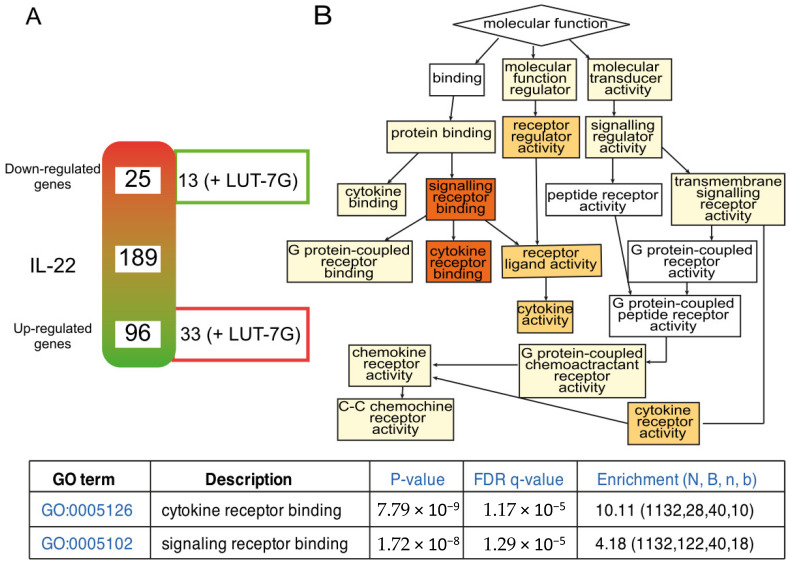
Inflammatory gene expression regulated by LUT-7G treatment, and clusterization analysis. (**A**) schematic representation of modulated genes after IL-22 treatment. The array contains in total 189 genes. On the right site of the gradient diagram, are represented the number of genes of which the treatment with LUT-7g reverts the effects of IL-22. (**B**) Clusterization function pathway obtained using the regulated genes. FDR is False Discovery Rate; N total number of genes; B total number of genes associated with a specific GO term; n is the flexible cut-off. Enrichment is defined as (b/n)/(B/N).

The cartoon shows that treatment with pro-inflammatory molecules (like IL-22) modify the expression of different genes, known to be modulated in psoriasis. Mostly all of them are downregulated (96) and a lower number (25) upregulated (Table S2). LUT-7G treatments is able to neutralize the IL-22 effects on some of the genes. Specifically, this happens for 13 of the 25 up-regulated genes, and for 33 of the 96 down-regulated genes (Figure 4A, Table S2). The analysis by clusterization using Go-terms, and the GoRilla algorithm (Figure 4, left panel) demonstrated the convergence of regulated genes to pathways of cytokine activity. The color (red is high) indicates the number of genes that converge in the specific cluster, the GO:0005126, GO:0005102 with confidence *p*-value are reported in the bottom of Figure 4. 

As previously stated, high confidence clusterization has been obtained in GO:0005126, that contain interesting genes connected also with vascular inflammation and development of atherosclerosis (Table S2). In this group, we found the IL23R, recently found increased in giant-cell arteritis (GCA) lesions and recognized to play central role in stimulating inflammatory and proliferative pathways relevant to GCA pathogenesis [42,43]. In addition, the inhibition of the toll-like receptor 9 (TLR9) present in the list has been recognized to promote a kind of cardiac inflammation using a mouse model of TLR9-deficient (TLR9-D) [44]. In fact, this is one of the genes repressed by IL-22, and which the expression is restored by the LUT-7G treatment. The flavone is also able to counteract the upregulation of IL-33. This cytokine is expressed in endothelial cells and it is known to drive endothelial inflammation [45], even if its role is not completely clarified. S100A7 is another important molecule modulated by the flavone. This protein is important not only in psoriasis physiology, but also in the processes of vascular remodeling following redox activation [46]. Although the two gene panels are different, some genes have been found significatively regulated in both panels like CXCR4, IL10RB, CCR6.

### 2.4. The Analysis of Metabolic Changes Induced by LUT-7G Show Reduction of Hydroxylated Medium Chain Fatty Acids and Hydroxylated Sterols

**Figure 5:**
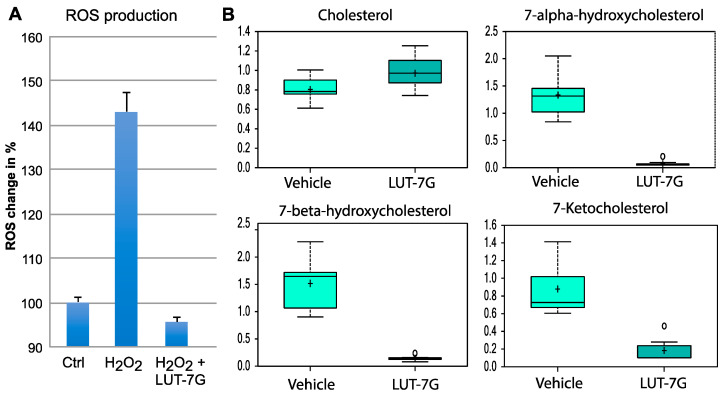
Oxidative stress and fatty acids hydroxylation analysis. (**A**) ROS production analysis, the results are presented in % units considering the control as 100%. (**B**) LUT-7G treatment affects cholesterol levels, which increase, and levels of hydroxylated species, which decrease dramatically. Data are shown as mean and standard deviation. Welch’s two-sample *t*-test was used to identify biochemicals that differed significantly between experimental groups. A *p* < 0.05 was considered significant (see Section 4).

Since we demonstrated that the STAT3 pathway is active and is inhibited by LUT-7G in HUVEC cells, we also investigated the possible role of the flavone in ROS production in these cells. We used HUVEC cultured cells treated with LUT-7G in the same conditions of the previous experiments. The results showed that this molecule is able to inhibit ROS production (Figure 5A), as in keratinocytes. The analysis of ROS production also demonstrated that the flavone retains high antioxidant properties, being able to counteract the production of oxygen reactive species induced by hydrogen peroxide. In fact, our tests performed on cultured HUVEC cells (Figure 5A) show that the treatment is able to restore the original ROS level of untreated cells, even if in the presence of the inducer. In fact, as it is visible in the graph, the treatment using hydrogen peroxide increases the formation of ROS (>>140%), while the treatment with LUT-7G, even in the presence of the hydrogen peroxide, is able to restore the basal oxidative stress condition in HUVEC cells. Inhibition of the STAT3 pathway and ROS production previously demonstrated in keratinocytes is retained also in HUVEC cells. It has been described in literature that the ROS-mediated reactions are responsible for oxysterols production like 7-ketocholesterol, 7a-hydroxycholesterol, and other hydroxylated fatty acids in different cell types like human aortic SMCs, HUVEC, and others with implication in atherosclerosis pathophysiology [14,47,48].

We used a metabolomic analysis performed in keratinocytes to analyze the modification of cholesterol and fatty acids hydroxylation general pathway mediated by LUT-7G. Analysis of metabolic changes induced by the treatment with the flavone showed a remodeling of both hydroxylated fatty acids medium chain, that of hydroxylated species of cholesterol. These changes are important since both of these classes of products are involved in lipid peroxidation and their oxidation products have been correlated to inflammatory signaling in the damaged artery, and the generation of atherosclerotic plaque [14,49]. As shown in Figure 5, the levels of cholesterols inside cells seem to be slightly increased after the treatment with LUT-7G, while the hydroxylated species, as 7-alpha-hydroxicholesterol, 7-beta-hydroxicholesterol, and 7-ketocholesterol are drastically reduced (*p* < 0.05). This information is important since these oxysterols are the major sterols found in human atherosclerotic lesions and presented in oxidized low-density lipoprotein (LDL), where they promote the vascular inflammatory response. Specifically, it was found that 7-ketocholesterol increased extracellular IL-6 protein expression by enhancing secretion from VSMC and that this release of IL-6 resulted being crucial in the onset of cardiovascular disease via its ability to induce adhesion molecules in the endothelium and vascular smooth muscle cells (VSMC) proliferation [50,51].

**Figure 6:**
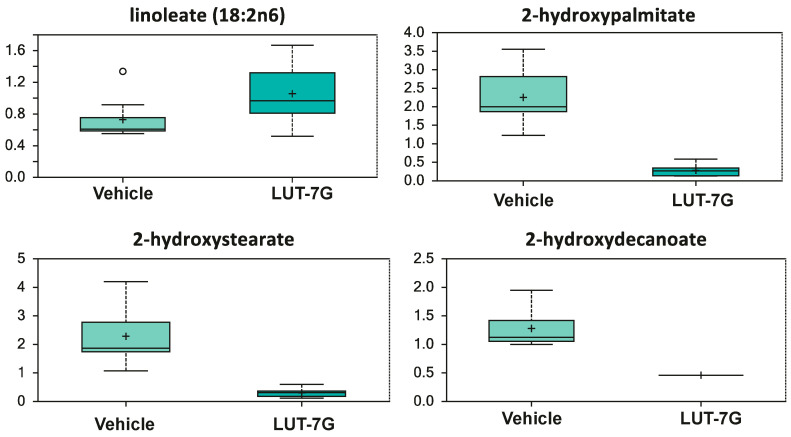
LUT-7G treatment affects linoleic acid levels, which increase, while its hydroxylated species is not detectable. Data are shown as mean and standard deviation. Welch’s two-sample *t*-test identified biochemical differences between experimental groups. A *p* < 0.05 was considered significant (see Section 4).

Continuing to look to hydroxylation phenomena, Figure 6 shows that in the samples treated with LUT-7G, there is an increase of linoleic acid, while its hydroxylated species is not detectable (*p* < 0.05). As it has been known, the dietary supplementation with linoleate has been associated with the reduction of cardiovascular risk [52]. Furthermore, although linoleate can be metabolized into arachidonic acid (AA), a PUFA precursor of eicosanoids which are a class of lipids including some with proinflammatory or prothrombotic-vasoconstrictor action, our previous analysis also showed the decrease of prostaglandin E2 [12], again leading to consider the treatment as anti-inflammatory. The reduction of hydroxylated species is relevant since the increased levels of these compounds are associated with pro-inflammatory cytokines production, vascular inflammation, and the development of cardiovascular diseases [53]. All together, these results demonstrate at the molecular level that LUT7-G shows anti-inflammatory properties with the regulation of metabolites important in endothelial and circulatory homeostasis. 

## 3. Discussion

Luteolin is one of the most common flavones with antioxidant, anticancer, anti-inflammatory, and antiapoptotic properties [9,10]. The antioxidant activity of luteolin and its glycosides have been evaluated and confirmed [54,55]. It has been observed that luteolin inhibits inflammatory responses induced by lipopolysaccharide (LPS) [10], tumour necrosis factor-α (TNF-α), and IL-6 in a dose-dependent manner. 

Particularly, STAT3 has been correlated to MMP secretion and induced epithelial-mesenchymal transition (EMT), and luteolin has been demonstrated to be able not only to inhibit STAT3 activity, but also the expression of metalloproteinase such as MMP2, MMP7, MMP9, and EMT [56]. Moreover, it has been also shown that luteolin is able to neutralize IL-6 downstream effects in keratinocytes [12] as well in a wide casuistry of tumour cell lines [56,57,58]. These data confirm that luteolin could play a role in the reduction of invasiveness of cancer cells through the deactivation of STAT3 and the reduction of IL-6 effects. Moreover, our previous results in the inhibition of glucose metabolism by LUT-7G indicate also the possibility for this flavone to counteract the Warburg effect typical of neoplastic cells, thus providing an additional tool in cancer therapy [13].

The anti-inflammatory activity of luteolin elicited by the regulation of mitogen-activated protein kinase (MAPKs) and its regulated transcription factors, has been also assessed. In fact, IL-1β-induced JNK and p38 activation were blocked by luteolin treatment. Similarly, IL-1β-induced activator protein-1 (AP-1) and nuclear factor-kappaB (NF-κB) activation were also blocked. These results provide evidence that luteolin counteracts the production of MMPs and cytokines by the inhibition of MAPKs (JNK and p38) and related transcription factors, like AP-1 and NF-κB [59,60]. In HUVEC cells, luteolin ameliorates TNF-α-induced oxidative stress and inflammation affecting the Nox4/ROS-NF-κB and MAPK pathways. These findings propose that luteolin may exert beneficial effects in treating vascular diseases associated with oxidative stress and inflammation [61].

In this work, we have investigated a possible anti-inflammatory capacity of LUT-7G in the endothelial district, to test if this molecule may be useful for the treatment of pathologies characterized by inflammatory processes. 

Our results demonstrated that the flavone is able to modify the physiology of endothelial cells by the modification of cell cycle parameters. Our findings on cell cycle analysis and scratch assay, in HUVEC cells treated with LUT-7G, highlight the anti-proliferative properties of this flavone. Interestingly, the modification of proliferation markers such as Ki67 and of vWF confirmed, respectively, the anti-proliferative and the pro-differentiating effects of LUT-7G in endothelial cells. Our experiments demonstrated that this treatment is able to inhibit the STAT3 pathway and ROS production also in endothelial cells, showing that its activity involves the same pathway in different cell systems.

In endothelial cells (HUVEC), the transcriptomic analysis of cytokines involved in the inflammation process showed that LUT-7G is able to repress a number of inflammatory cytokines and/or their specific repressors, as well induce anti-inflammatory molecules (IL10RB, ICEBERG, Table 1). This action has also been demonstrated using a psoriasis gene panel in human keratinocytes in which LUT7-G showed the ability to neutralize the inflammation psoriatic process induced by IL-22, thus demonstrating a wide anti-inflammatory activity, mostly independent from the cell type model used. This is really interesting since a direct link has been demonstrated between inflammatory diseases of the skin, associated with chronic inflammation such as psoriasis, with atherosclerosis, dyslipidaemia, and mortality for cardiovascular diseases [39,40,41].

Here, we also demonstrated another important molecular action of LUT7-G in the inhibition of the production of oxysteroles and hydroxylated fatty acids. To define the general pathway of modification of these classes of lipids, we used a keratinocyte cell model in which LUT-7G is also able to counteract ROS generation and to inhibit STAT3 signaling. These lipids species are involved in inflammatory processes of skin, in vascular inflammation, as well in atherosclerosis pathophysiology. In particular, our metabolomic analysis clarified how, in the cells treated with LUT-7G, the levels of the hydroxylated species of cholesterol decrease drastically. This is important because, in literature, data are present showing that these reactive cholesterol species are strongly involved in atherogenesis [14]. The effects of LUT-7G on lipid hydroxylation in our experiment also showed the increase of linoleic acid levels while its hydroxylated species show a very low concentration after the treatment. Hydroxylated species of cholesterols, such as 7-alpha-hydroxicholesterol, 7-beta-hydroxicholesterol, and 7-ketocholesterol are drastically reduced upon the treatment with LUT-7G and they have been described as biomarkers of oxidative stress [62]. Furthermore, 7-beta-hydroxycholesterol is a very hydrophobic molecule, present in atheromatous plaques and in the plasma of atherosclerotic patients [63], and it has been shown to enhance or to be enhanced by oxidative stress [64]. This is important under the consideration that in our HUVEC system, LUT-7G reduces ROS production. [65].

Atherosclerosis, such as psoriasis, is characterized by a state of chronic inflammation of vascular wall. This condition is sustained by pro-inflammatory cytokines that recruits immune cells and platelets, which in turn secrete other pro-inflammatory molecules. The oxidative stress associated with the inflammation affects endothelial and vascular function, and contributes to vascular disease. In these states of inflammation, NO production is impaired in the vasculature leading to endothelial dysfunction and a predisposition to CAD. In this context, the suppression of pro-inflammatory cytokine responses via targeting the JAK/STAT3 pathway, and the inhibition of ROS production could give advantages for a potential therapeutic strategy to counteract vascular inflammation and reduce the cardiovascular risk [6,8]. In fact, it has been also demonstrated that luteolin can suppress also oxLDL-induced inflammation via the inhibition of STAT3 activation, and that it is able to counteract the development and progression of atherosclerosis in ApoE−/− mice with HFD [19]. In this mouse system, the administration of luteolin decreased macrophages infiltration and mRNA expression of inflammatory factors like ICAM-1, VCAM-1, TNF-α, and IL-6. 

In our study, we found also that LUT-7G has a direct effect in lowering the generation of ROS, as we proved in our vascular cell system. Thus, LUT-7G could produce cardiovascular benefits, through the demonstrated inhibition of STAT3, the downregulation of target genes involved in inflammation, and by inhibiting ROS production in endothelial cells. For example, in the HUVEC system, LUT-7G reduces the expression of IL-1β, which has been demonstrated to be a clinically applicable intervention to improve the cardiovascular outcomes, leading to a new use of anti-inflammatory therapies for atherosclerosis [31]. 

Taking together, these findings report for the first time, a molecular signature of the previous epidemiological studies, showing the possible beneficial effects of luteolin on the cardiovascular system [66]. Interestingly, in this study, we used the specific compound LUT-7G, the glycosylated isoform present in vegetables. Our experiments demonstrate also its potential cellular uptake, since we retrieved in cell-treated cytoplasm, both glycosylated and not conjugated luteolin molecular species (Figure S2).

Indeed, this set of data candidate LUT-7G as a new pharmacological molecule that can be evaluated in the treatment of inflammatory diseases, in the skin as well in CVD.

## 4. Materials and Methods

### 4.1. The Cell Culture

Human epidermal keratinocytes (neonatal) (HEKn) (Cascade, Invitrogen) were cultured in Epilife medium with human keratinocyte growth supplements (HKGS) added (Cascade). HEKn were grown at 37 °C in a humidified incubator with 5% CO_2_. HEKn cells were seeded on collagen-coated dishes and kept constantly subconfluent to avoid triggering of differentiation. At each passage, cells were harvested, counted, seeded, and collected to extract RNA and proteins. Cells were induced to differentiate by adding 1.2 mM CaCl2 to the culture medium for different time period (2, 3, or 6 days). Otherwise, terminal differentiation of keratinocyte cultures was achieved also by growing cells at 100% of confluence and, thus, keeping them in culture for other 4 days without addition of growth factors in medium. LUT-7G (Sigma Aldrich, St. Louis, MO, USA) was dissolved in DMSO, and stored at +4 °C protected from light. It was used at a final concentration of 0.01% *v*/*v* of DMSO in media. IL-22 and IL-6 (R&D Systems, Minneapolis, MN, USA) were added to keratinocyte cultures at a final concentration of 50 ng/mL.

Human umbilical vein endothelial cells (HUVEC) were cultured in EBMTM endothelial cell growth basal medium (LONZA, Walkersville, MD, USA) with EGMTM-2 growth factors (LONZA, Walkersville, MD, USA) added. HUVEC were maintained at 37 °C in a humidified incubator with 5% CO_2_. All experiments were performed at p4 passage. Cells were treated with LUT-7G 20 uL in medium and harvest after 48 h of treatment. Control cells were grown in medium with a final concentration of 0.01% of DMSO and harvest after 48 h. In the scratch assay, the same culture conditions were maintained for treated and control cells. Scratch was done after 24 h of LUT-7G treatment and all cells (control/treated) were acquired every 4 h with IncuCyte S3 Live-cell analysis system (Bioscience, Napa, CA, USA). Time course: 0–4 h, 8–12 h, 16–20 h.

### 4.2. Metabolomics Analysis

Cell were harvested at passage 3 after 3 days of treatment. Control cells were grown with a final concentration in medium of 0.01% of DMSO (vehicle used to dissolve the flavone), and treated cells were grown with the addition of LUT-7G 20 µM in medium. For each condition, 9 replicates were obtained, with 10 × 10^6^ cells each. Samples were immediately stored at −80 °C and, at the time of analysis, were extracted and prepared for analysis using a standard metabolic solvent extraction method. The extracted samples were split into equal parts for analysis by gas chromatography/mass spectrometry (GC/MS) or liquid chromatography/mass spectrometry (LC/MS/MS) platforms. Additionally, included were several technical replicate samples created from a homogeneous pool containing a small amount of all study samples. The LC/MS portion of the platform was based on a waters ACQUITY UPLC and a Thermo-Finnigan LTQ mass spectrometer. The sample extract was split into 2 aliquots, dried, and then reconstituted in acidic or basic LC-compatible solvents, each of which contained 11 or more injection standards at fixed concentrations. One aliquot was analyzed using acidic positive ion optimized conditions and the other using basic negative ion optimized conditions in 2 independent injections using separate dedicated columns. Extracts reconstituted in acidic conditions were gradient eluted using water and methanol, both containing 0.1% formic acid, while the basic extracts, which also used water/methanol, contained 6.5 mM ammonium bicarbonate. The MS analysis alternated between MS and data-dependent MS2 scans using dynamic exclusion. The samples destined for GC/MS analysis were re-dried under vacuum desiccation for a minimum of 24 h prior to being derivatized under dried nitrogen using bistrimethyl-silyl-triflouroacetamide (BSTFA). The GC column was 5% phenyl and the temperature ramp is from 40 to 300 °C in a 16 min period. Samples were analyzed on a Thermo-Finnigan Trace DSQ fast-scanning single-quadrupole mass spectrometer using electron impact ionization. The instrument was tuned and calibrated for mass resolution and mass accuracy on a daily basis. The information output from the raw data files was automatically extracted.

For ions with counts greater than 2 million, an accurate mass measurement could be performed. Accurate mass measurements could be made on the parent ion as well as fragments. The typical mass error was less than 5 ppm. Ions with less than 2 million counts require a greater amount of effort to characterize. Fragmentation spectra (MS/MS) were typically generated in a data-dependent manner, but if necessary, targeted MS/MS could be employed, such as in the case of lower level signals. Compounds were identified by comparison with library entries of purified standards or recurrent unknown entities. Identification of known chemical entities was based on comparison with metabolic library entries of purified standards. The combination of chromatographic properties and mass spectra gave an indication of a match to the specific compound or an isobaric entity. Instrument variability was determined by calculating the median relative standard deviation (RSD) for the internal standards that were added to each sample prior to injection into the mass spectrometers. Overall process variability was determined by calculating the median RSD for all endogenous metabolites (i.e., non-instrument standards) present in 100% of the matrix samples, which are technical replicates of pooled client samples. The metabolic analysis comprises a total of 279 compounds named biochemicals. Following imputation of any missing values present with the minimum observed value for each compound, normalization to Bradford protein concentration and log transformation of median scaled data. Welch’s two-sample *t*-test was used to identify biochemicals that differed significantly between experimental groups. A summary of the numbers of biochemicals that achieved statistical significance (*p* ≤ 0.05), as well as those approaching significance (0.05 < *p* < 0.10), is shown below. 

An estimate of the false discovery rate (*q*-value) is calculated to take into account the multiple comparisons that normally occur in metabolomic-based studies. For example, when analyzing 200 compounds, we would expect to see about 10 compounds meeting the *p* ≤ 0.05 cut-off by random chance. The *q*-value describes the false discovery rate; a low *q*-value (*q* < 0.10) is an indication of high confidence in a result. While a higher *q*-value indicates diminished confidence, it does not necessarily rule out the significance of a result.

### 4.3. RNA Extraction and Quantitative Real-Time

RT-PCR. MirVana miRNA isolation kit (Ambion, Austin, TX, USA) was used for total RNA extraction from keratinocytes, The amount of 1 μg total RNA has been used for reverse transcription using the IScript Advanced CDNA synthesis (Biorad, code 1725037) while real-time PCR was performed using SSOADV UNIVER SYBR GRN qPCR Master Mix (Biorad, code 1725270). The expression of genes involved in the psoriasis pathway was detected using the PSORIASIS T1-4 H384 plates on a CFX384 Touch Real-Time PCR System (Biorad, code 10038559, and 1855485). All procedures were performed using the manufacturer’s instructions. Each gene expression profile was defined from the threshold cycle (Ct), and relative expression levels were calculated by using the run-file and software provided by the manufacturer (Biorad, Hercules, CA, USA), by using the internal controls and statistical tools provided. (Run file and primer validation data can be found on: https://www.bio-rad.com/it-it/prime-pcr-assays/predesigned-plate/sybr-green-psoriasis-h384). 

Pro-inflammatory cytokine gene analysis in HUVEC cells, was performed by RT Profiler PCR Array System (1022A, Bioscience Corporation) according to the manufacture’s instruction. 

### 4.4. Western Blotting Analysis

For Western blotting analysis, HUVEC lysate was resolved on SDS-polyacrylamide gel and blotted onto Hybond PVDF membrane (G&E Healthcare, Waukesha, WI, USA). The following antibodies: rabbit polyclonal anti-STAT3 C-20 (SantaCruz, dilution 1:500, prepared in blocking buffer; 5% milk in PBS 0.1% tween) and mouse monoclonal anti-β-actin (A5441 Sigma, dilution 1:5000 prepared in PBS 0.1% tween). β-actin was used as a loading control.

### 4.5. Confocal Analysis

HUVEC cells were fixed in formalin 4% and permeabilized with Triton X 0.1% for 10 min. Cells were incubated in blocking buffer PBS 1x + 2% GS for 1 h. Following primary antibodies, rabbit polyclonal anti-STAT3 C-20 (SantaCruz, dilution 1:500), rabbit anti KI-67 (9129S, cell signaling, dilution 1:200), mouse anti-vWF (Takara, Shiga, Japan, dilution 1:200). Following secondary antibodies: anti mouse ALEXA FLUOR^®^ 488 and anti-rabbit ALEXA FLUOR^®^ 488 (Invitrogen, Carlsbad, CA, USA, dilution 1:1000, prepared in blocking buffer). Cytoskeleton was stained with ALEXA FLUOR^®^ 568 Phalloidin (dilution 1:1000) in PBS 1x while nuclear staining was performed by DAPI (dilution 1:1000). All antibodies were prepared in blocking buffer and all steps were performed at room temperature. Cells were mounted using the Prolong Antifade kit (Invitrogen) and analyzed with a confocal laser microscope NIKON Eclipse Ti). Signal detection was performed using NIS elements AR4.00.04 software (Nikon, Tokyo, Japan).

### 4.6. ROS Detection and Cell Cycle Analysis

HUVEC cells and medium were harvested using trypsin 1× and centrifuged at 1000× *g* rpm for 10 min. Pellet was suspended in PBS 1x-CM-H2DCFDA (stock 50 µg, dissolved in DMSO, Life Technologies, Carlsbad, CA, USA) to a final concentration of 10 µM. Cells were incubated at room temperature for 10 min. For cell cycle, harvested cells were washed in PBS 1x and fixed with 1:1 PBS and methanol-acetone (4:1 (*v*/*v*) solution at −20 °C). HUVEC cells were treated with 50 µL of a solution of 13 Kunitz/mL RNaseA at 37 °C for 15 min and stained with 50 mg/L propidium iodide (Sigma) overnight at for 4 °C. ROS detection was performed using Beckman Coulter CytoFLEX with CytExpert software. Cell cycle was assed using ModFit software (Becton Dickinson, Franklin Lakes, NJ, USA).

## Figures and Tables

**Table 1:** Regulation of chemokines in HUVEC cells treated with LUT-7G. In the table, are reported the fold over controls, calculated as 2^∆∆Ct^, respect to the basal value obtained in untreated cells. The asterisks refer to values of receptors common to different chemokines. * Fold-L/Fold-R is yet specified for each ligand/receptor. (not shown) in this case, the gene has been not analyzed since the primers were not available in the assay.

Ligand	Fold−L	Receptor	Fold−R
CCL1	−2.2387405	CCR8	−3.3123513
CCL11	−4.9576884	CCR3	−10.864165
CCL24	−2.258471	CCR3	−10.864165
CCL13	−4.8259756	CCR3- CCR2B	*
CCL2	−5.7795846	CCR2	−9.3664778
CCL15	−2.8973017	CCR1	−1.4173113
CCL7	−7.5063997	CCR1-CCR2-CCR3	*
CCL19	−10.563739	CCR7	−2.3949673
CCL21	−2.8918255	CCR7	−2.3949673
CCL25	−1.9211331	CCR9	(not shown)
CCL3	−10.469666	CCR1-CCR4-CCR5	*
CCL8	−3.1094217	CCR1-CCR2-CCR3	*
CXCL12	−8.7263689	CXCR4	(not shown)
Fractalkine	(not shown)	CX3CR1	−2.7853406
CXCL1	−2.0222758	CXCR2	(not shown)
CXCL5	−1.2044205	CXCR2	(not shown)
IL13-IL4	(not shown)	IL13RA1	−1.6702992
IL1B	−3.0131935	IL1R1-IL1R2	(not shown)
IL1F6 (IL36A)	−47.877184	IL1RL2-IL36R	(not shown)
CXCL10	−44.252749	CXCR3	(not shown)
CCL15	−2.8973017	CCR1-CCR3	*
B4 Leukotriene	(not shown)	LTB4R	−3.0392471
IL10	(not shown)	IL10RB	1.87
ICEBERG (CARD18)	8.2		

## Data Availability

All data are reported in the manuscript and in the Supplementary Material.

## Supplementary Materials

The following are available online at https://www.mdpi.com/1422-0067/22/3/1321/s1, Figure S1: Modeling of STAT3/LUT-7G binding; Figure S2: Luteolin content in treated cells; Table S1: Regulated cytokine genes; Table S2: regulated genes used for clusterization in Figure 4.
