# Supplementary material for: Anti-Inflammatory and Proliferative Properties of Luteolin-7-O-Glucoside
Source: Int J Mol Sci. 2021 Jan 28;22(3):1321. doi: 10.3390/ijms22031321 (PMC7865871; doi:10.3390/ijms22031321)
Supplement: Supplementary file 1 [file ijms-22-01321-s001.zip › Figure S1.pdf]

Figure S 1

A

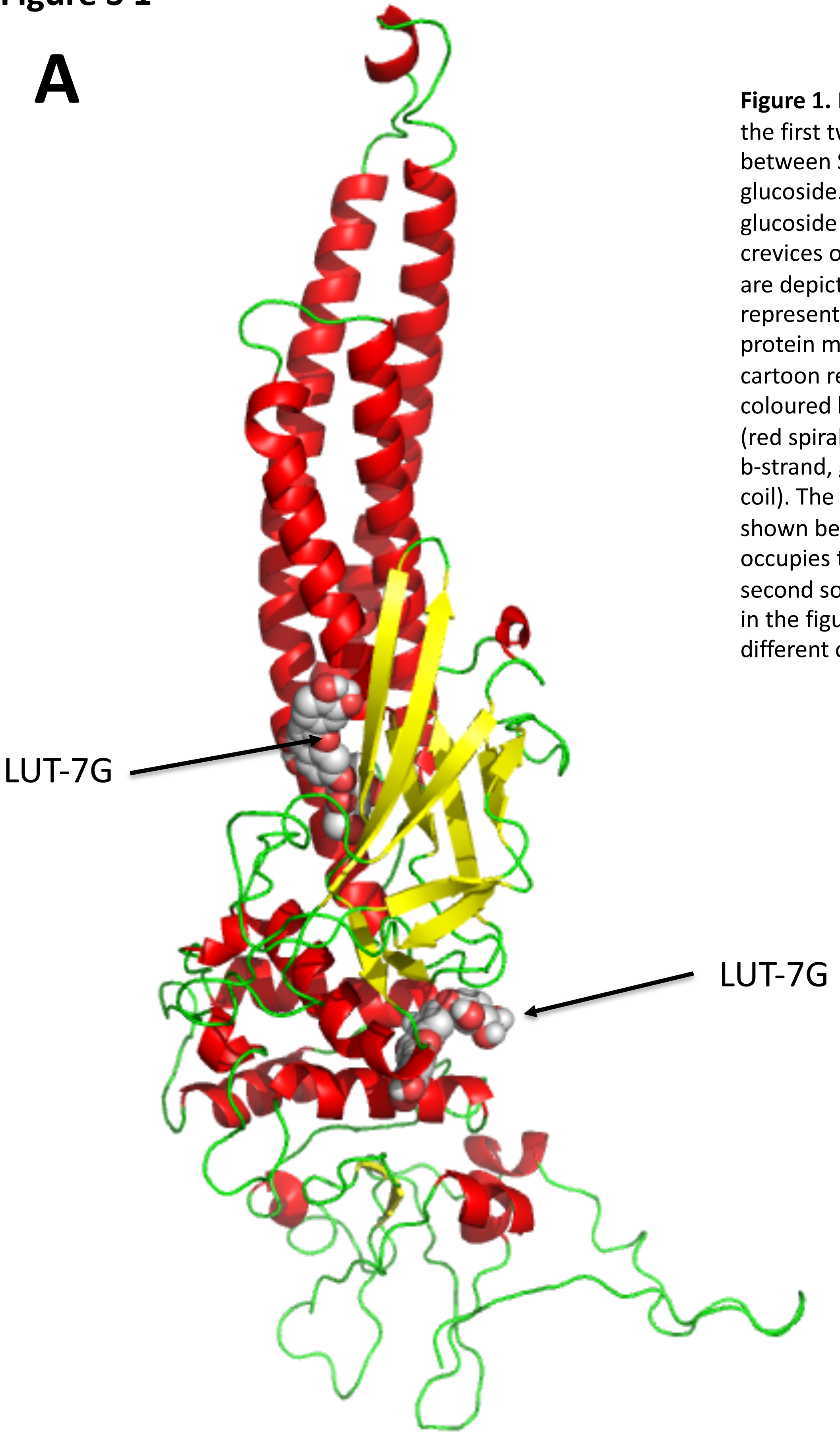

Figure 1. Docking complex of the first two clustered solutions between STAT3 and luteolin-7-glucoside. The luteolin-7-glucoside molecules, hosted on crevices on the STAT3 surface, are depicted by spacefill representations while the protein model is shown as a cartoon representation coloured by secondary structure (red spiral a-helix, yellow arrow b-strand, green tube random coil). The third solution is not shown because luteolin occupies the same site as the second solution (the one below in the figure), but with a different conformation

Simulation methods

Model building

In the absence of the human STAT3 X-ray structure, the crystal structure of the phosphorylated STAT3 from *Mus musculus*, deposited in the Protein Data Bank (PDB, http://www.rcsb.org/pdb) with PDB ID code 1BG1 (Becker et al., 1998), has been used as a template to achieve the model structure. The model has been generated using the SWISS-MODEL protein modelling tool (swissmodel.expasy.org) (Biasini et al., 2014) and has been used as a receptor for the molecular docking analysis. Due to the high identity and coverage between the two sequences (100% and 64%, respectively) the overall structure is well identified by the modelling procedure making the structure appropriate for the simulative docking analysis.

Molecular Docking procedure

Protein-ligand molecular docking has been used to predict the complexes between the human STAT3 model and luteolin-7-glucoside. The docking simulations have been executed using the AutoDock Vina 1.1.2 program (Trott and Olson, 2010), through the AutoDock/Vina PyMOL plugin (http://wwwuser.gwdg.de/~dseelig/adplugin.html) (The PyMOL Molecular Graphics System Version 1.5.0.4. Schrödinger, LLC; Seeliger and de Groot, 2010). The luteolin-7-glucoside SDF file, downloaded from the PubChem compound database (https://pubchem.ncbi.nlm.nih.gov), has been converted into mol2 file and completed with missing hydrogen atoms using the Open Babel program (O'Boyle et al., 2011). In a first docking simulation, in the absence of the human enzyme structure, the STAT3 model has been used as receptor. The simulation has been performed using the Genetic Algorithm with local gradient optimization (Trott and Olson, 2010). The docking box (dimensions x = 131.25; y = 105.00; z = 168.75 Å) contains the whole STAT3 modelled monomer to evaluate the presence of possible binding sites over the protein surface.

Simulative results

The molecular docking analysis detects a binding energy of over -8.0 kcal/mol for the first three clustered solutions in crevices on the STAT3 surface and of over -7.0 kcal/mol for the remaining seven solutions, confirming that luteolin-7-glucoside may block the activity of STAT3. Computational modelling shows that luteolin-7-glucoside could bind to the SH2 domain of STAT3, albeit with a less intense binding energy solution (-7.0 kcal/mol), suppressing the dimerization mechanism.

References

Nkansah E, Shah R, Collie GW, Parkinson GN, Palmer J, Rahman KM, Bui TT, Drake AF, Husby J, Neidle S, Zinzalla G, Thurston DE, Wilderspin AF. Observation of unphosphorylated STAT3 core protein binding to target dsDNA by PEMSA and X-ray crystallography. FEBS Lett. 2013 Apr 2;587(7):833-9.

Becker S, Groner B, Müller CW. Three-dimensional structure of the Stat3beta homodimer bound to DNA. Nature. 1998 Jul 9;394(6689):145-51. PubMed PMID: 9671298.

De Lano W.L., The PyMOL Molecular Graphics System, DeLano Scientific. San Carlos, CA, USA (2002).

Marco Biasini, Stefan Bienert, Andrew Waterhouse, Konstantin Arnold, Gabriel Studer, Tobias Schmidt, Florian Kiefer, Tiziano Gallo Cassarino, Martino Bertoni, Lorenza Bordoli, Torsten Schwede. (2014). SWISS-MODEL: modelling protein tertiary and quaternary structure using evolutionary information. Nucleic Acids Research; (1 July 2014) 42 (W1): W252-W258; doi: 10.1093/nar/gku340.

O'Boyle N.M., Banck M., James C.A., Morley C., Vandermeersch T., Hutchison G.R. (2011) Open Babel: An open chemical toolbox. J. Cheminform. 3:33.

Pettersen E.F., Goddard T.D., Huang C.C., Couch G. S., Greenblatt D.M., Meng E.C. and Ferrin T.E. (2004) UCSF Chimera - A visualization system for exploratory research and analysis. J. Comput. Chem. 25, 1605-1612.

Seeliger D., de Groot B.L. Ligand docking and binding site analysis with PyMOL and Autodock/Vina., Journal of Computer-Aided Molecular Design. 24 (2010) 417–422.

The PyMOL Molecular Graphics System Version 1.5.0.4. Schrödinger, LLC.

Trott O., Olson A.J., AutoDock Vina: improving the speed and accuracy of docking with a new scoring function, efficient optimization, and multithreading. (2010) Journal of Computational Chemistry. 31, 455–461.
