# Supplementary material for: Anti-Inflammatory and Proliferative Properties of Luteolin-7-O-Glucoside
Source: Int J Mol Sci. 2021 Jan 28;22(3):1321. doi: 10.3390/ijms22031321 (PMC7865871; doi:10.3390/ijms22031321)
Supplement: Supplementary file 1 [file ijms-22-01321-s001.zip › Table S1.pdf]

TABLE S1 In table are reported all genes amplified with significative regulat

RefSeq	Symbol	DDct	Fold	Regulation	p-value <0.05	
NM_014440	IL1F6	5,5812664	0,02088678	-47,877184	yes	
NM_001837	CCR3	3,44150543	0,09204573	-10,864165		0,056
NM_006274	CCL19	3,40104866	0,09466345	-10,563739	yes	
NM_002983	CCL3	3,38814354	0,09551403	-10,469666	yes	
NM_000648	CCR2	3,22750664	0,10676372	-9,3664778		0,057
NM_000609	CXCL12	3,12538147	0,1145952	-8,7263689	yes	
NM_006273	CCL7	2,90812111	0,13321966	-7,5063997	yes	
NM_002982	CCL2	2,53096581	0,17302282	-5,7795846	yes	
NM_012275	IL1F5	2,40226746	0,18916703	-5,2863335	yes	
NM_002986	CCL11	2,30966759	0,20170691	-4,9576884	yes	
NM_005408	CCL13	2,27082062	0,20721199	-4,8259756	yes	
NM_005201	CCR8	1,72785568	0,30190035	-3,3123513	yes	
NM_005623	CCL8	1,63664627	0,32160321	-3,1094217	yes	
NM_181657	LTB4R	1,60371399	0,32902885	-3,0392471	yes	
NM_000576	IL1B	1,59129333	0,3318738	-3,0131935	yes	
NM_032965	CCL15	1,53470993	0,34514873	-2,8973017	yes	
NM_002989	CCL21	1,53198051	0,34580233	-2,8918255	yes	
NM_001337	CX3CR1	1,47785378	0,35902251	-2,7853406	yes	
NM_001090	ABCF1	1,40206528	0,37838708	-2,6427964	yes	
NM_001838	CCR7	1,26000595	0,41754224	-2,3949673	yes	
NM_002991	CCL24	1,17534637	0,44277744	-2,258471	yes	
NM_002981	CCL1	1,1626873	0,44667973	-2,2387405	yes	
NM_001101	ACTB	1,15596771	0,44876507	-2,2283374	yes	
NM_002987	CCL17	1,09645462	0,46766436	-2,1382857	yes	
NM_000194	HPRT1	1,08899498	0,47008874	-2,1272579	yes	
NM_001511	CXCL1	1,01597977	0,49449239	-2,0222758	yes	
SA_00104	RTC	0,99015999	0,50342194	-1,9864053	yes	
NM_000877	IL1R1	0,97197151	0,50980891	-1,9615193	yes	
NM_005624	CCL25	0,94195747	0,52052614	-1,9211331	yes	
NM_001560	IL13RA1	0,74010658	0,59869512	-1,6702992	yes	
NM_000582	SPP1	0,73706436	0,59995893	-1,6667808	yes	
NM_019009	TOLLIP	0,71012878	0,61126557	-1,6359501	yes	
NM_000064	C3	0,67518997	0,62624975	-1,596807	yes	
NM_002415	MIF	0,63177109	0,64538364	-1,549466	yes	
NM_173161	IL1F10	0,57015228	0,67354569	-1,4846803	yes	
SA_00104	RTC	0,5293293	0,69287677	-1,4432581	yes	
NM_001295	CCR1	0,50315666	0,7055613	-1,4173113		0,1246
NM_000572	IL10	0,33763695	0,79133641	-1,2636851	yes	
NM_005283	XCR1	0,30703735	0,80829994	-1,2371645	yes	
NM_000074	CD40LG	0,29679108	0,81406106	-1,2284091	yes	
NM_002994	CXCL5	0,26833916	0,83027482	-1,2044205		0,82
NM_002341	LTB	0,22827911	0,85365254	-1,1714368	yes	
NM_002188	IL13	0,00016403	0,99988631	-1,0001137	yes	
NM_004048	B2M	0	1		yes	
NM_005064	CCL23	-0,0061283	1,00425686	1,00425686	yes	

NM_173205	IL1F7	-0,0591297	1,0418371	1,0418371	yes
NM_001735	C5	-0,0976505	1,07002947	1,07002947	yes
NM_004757	SCYE1	-0,1027546	1,0738218	1,0738218	yes
NM_006419	CXCL13	-0,1665478	1,12236955	1,12236955	yes
SA_00103	PPC	-0,3123531	1,2417314	1,2417314	yes
NM_002046	GAPDH	-0,3188286	1,24731736	1,24731736	yes
NM_004590	CCL16	-0,3468838	1,27181054	1,27181054	yes
NM_000595	LTA	-0,4533558	1,36922144	1,36922144	yes
NM_000577	IL1RN	-0,5293217	1,44325045	1,44325045	yes
NM_173178	IL1F8	-0,5861187	1,5012026	1,5012026	yes
NM_000575	IL1A	-0,5952702	1,51075546	1,51075546	yes
SA_00103	PPC	-0,6772728	1,59911401	1,59911401	yes
NM_004367	CCR6	-0,6992035	1,62360815	1,62360815	yes
NM_000628	IL10RB	-0,7097416	1,63551115	1,63551115	yes
NM_000594	TNF	-0,7638912	1,69806445	1,69806445	yes
NM_012423	RPL13A	-0,7862797	1,72462139	1,72462139	yes
NM_000879	IL5	-0,8671341	1,82403586	1,82403586	yes
NM_007293	C4A	-0,9030132	1,86996754	1,86996754	yes
NM_002089	CXCL2	-0,903965	1,87120159	1,87120159	yes
NM_006641	CCR9	-0,9751682	1,96587042	1,96587042	yes
NM_005409	CXCL11	-1,0747337	2,10633329	2,10633329	yes
NM_004887	CXCL14	-1,0823326	2,11745691	2,11745691	yes
NM_013278	IL17C	-1,1250477	2,18108755	2,18108755	yes
NM_002993	CXCL6	-1,1971207	2,29281613	2,29281613	yes
NM_006072	CCL26	-1,2520542	2,3818032	2,3818032	yes
NM_000605	IFNA2	-1,2796478	2,42779705	2,42779705	yes
NM_000584	IL8	-1,2816257	2,43112782	2,43112782	yes
SA_00103	PPC	-1,2923317	2,44923583	2,44923583	yes
NM_002985	CCL5	-1,6264343	3,08748971	3,08748971	yes
NM_002186	IL9R	-1,8166695	3,52267032	3,52267032	yes
SA_00105	HGDC	-1,8822346	3,68645609	3,68645609	yes
NM_000590	IL9	-2,1250935	4,36231352	4,36231352	yes
NM_005194	CEBPB	-2,1470165	4,42910907	4,42910907	yes
NM_002090	CXCL3	-2,7298298	6,63377366	6,63377366	yes
NM_021571	ICEBERG	-2,8145866	7,0351766	7,0351766	yes
NM_000567	CRP	-3,655405	12,6004648	12,6004648	yes
NM_019618	IL1F9	-3,6586285	12,6286495	12,6286495	yes
NM_000579	CCR5	-3,7510548	13,4641828	13,4641828	yes
NM_002984	CCL4	-5,235136	37,6645668	37,6645668	yes
NM_005508	CCR4	-6,1016541	68,6721893	68,6721893	yes
