# Supplementary material for: Anti-Inflammatory and Proliferative Properties of Luteolin-7-O-Glucoside
Source: Int J Mol Sci. 2021 Jan 28;22(3):1321. doi: 10.3390/ijms22031321 (PMC7865871; doi:10.3390/ijms22031321)
Supplement: Supplementary file 1 [file ijms-22-01321-s001.zip › Table S2.pdf]

Data as relative expression
Only significative genes have been reported

DOWNREGULATED

GENE	IL22 (-) Regulation
KRT10	-10,22
AQP9	-6,88
CCL22	-5,69
RNASE7	-4,59
NTRK2	-4,25
RARRES2	-3,72
TYRP1	-3,16
TAC1	-3,00
PRKCB	-2,84
CXCR4	-2,84
EREG	-2,81
CD52	-2,73
CEBPA	-2,64
IL12RB1	-2,37
LGALS4	-2,36
IFRD1	-2,34
IL1B	-2,27
DRD5	-2,18
IL6	-2,17
CAMP	-2,13
ZNF43	-2,11
DUSP1	-2,08
CCR1	-2,07
IL12A	-2,05
MMP9	-1,99
HBEGF	-1,94
PPARD	-1,92
IL18	-1,91
IL10RA	-1,80
IL1A	-1,79
KLF4	-1,77
LGALS1	-1,76
CCL5	-1,74
IL36G	-1,73
CLDN23	-1,72
ZFP36	-1,68
HIST1H1C	-1,62
IL8	-1,62
SATB1	-1,62
IL15	-1,61
CCNE1	-1,58
SERPINB3	-1,57
LGALS3	-1,56
MAFF	-1,55
CXCL2	-1,54
NFAT5	-1,51
KRT6B	-1,48
ADM	-1,47
CLK1	-1,46
IL1RN	-1,46
HSPA1A	-1,43
HBB	-1,42
TLR9	-1,42
CDH4	-1,40
CCR6	-1,39
HSPA1B	-1,39
TGFA	-1,38
CEBPB	-1,38
FOXP3	-1,37
DPP4	-1,35
CREM	-1,33
CDKN1A	-1,33
RBBP7	-1,33
PTCH1	-1,31
MALT1	-1,29
GAPDH	-1,28
IL10RB	-1,28
PTHLH	-1,27
NOTCH2	-1,27
LGALS8	-1,27
CLIC1	-1,26
PMF1	-1,26
TNF	-1,26
CDH1	-1,24
BSG	-1,24
REL	-1,23
MAP2K1	-1,23
IL7R	-1,23
ADORA2B	-1,22
CFLAR	-1,22
HLA-C	-1,22
EPHA2	-1,22
ELAVL1	-1,21
HES1	-1,20
TJP1	-1,20
CXCL11	-1,19
SERPINB6	-1,19
TPMT	-1,19
BTF3	-1,19
TCEB3	-1,18
KRT5	-1,18
RUNX1	-1,18
AREG	-1,17
RPL35A	-1,17

DOWNREG-Partially Reverted

Gene	IL-22	IL22+Lut	
KRT10	-10,22446	-1,43537	
RNASE7	-4,59319	-1,3256	
NTRK2	-4,25193	-1,57289	
TYRP1	-3,1568	1,07081	
PRKCB	-2,84323	1,54631	
CXCR4	-2,84265	2,50617	
IL12RB1	-2,37313	1,32389	
LGALS4	-2,35633	1,06707	
ZNF43	-2,10745	1,15024	
IL10RA	-1,80277	-0,25803	
HIST1H1C	-1,62243	1,407	
SATB1	-1,62043	1,47078	
LGALS3	-1,56426	1,02337	
NFAT5	-1,50761	1,06318	
ADM	-1,46506	1,49276	
CLK1	-1,46238	1,48809	
HSPA1A	-1,4269	1,21142	
HBB	-1,42189	2,26649	
CCR6	-1,39115	3,00321	
HSPA1B	-1,38522	1,2133	
CREM	-1,33452	1,31962	
PTCH1	-1,30915	1,62735	
MALT1	-1,2897	1,04492	
IL10RB	-1,27835	1,67595	
KLF4	-1,77	-1,41369	

UPREG-Partially Reverted

Gene	IL-22	IL22+Lut	
PTGS2	1,69	0,85	
DEFB1	1,83	0,98	
TNFSF11	1,84	0,90	
S100A8	2,02	1,24	
TLR2	2,02	1,18	
IL33	2,37	0,24	
IL23R	3,15	1,32	
VNN3	4,04	2,07	
SPP1	4,42	1,65	
CLEC7A	8,12	3,20	
S100A7	11,91	1,77	
DEFB4A	185,12	36,94	
TLR9	-1,4199	1,40	

UPREGULATED

Target	IL22 (+) Regulation
IL10	1,30
TP63	1,30
TLR3	1,32
KRT6A	1,35
LYN	1,37
VNN2	1,37
CXCR3	1,38
CCL20	1,40
TSLP	1,47
TNFSF10	1,48
PTGS2	1,49
CLDN4	1,55
GLI1	1,57
DEFB1	1,61
TNFSF11	1,62
S100A8	1,78
TLR2	1,78
PTGES	1,98
IL33	2,37
IL23R	3,15
VNN3	4,04
SPP1	4,42
CLEC7A	7,16
S100A7	10,50
DEFB4A	163,08
TLR9	0,80
