# Supplementary figures and images for: Anti-Inflammatory and Proliferative Properties of Luteolin-7-O-Glucoside
Source: Int J Mol Sci. 2021 Jan 28;22(3):1321. doi: 10.3390/ijms22031321 (PMC7865871; doi:10.3390/ijms22031321)

**luteolin-7-O-glucoside**

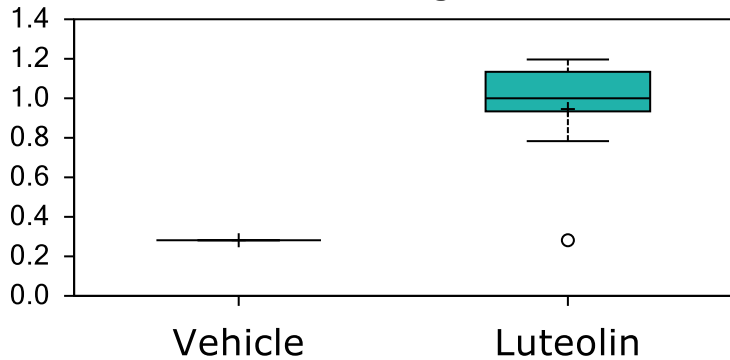

**luteolin**

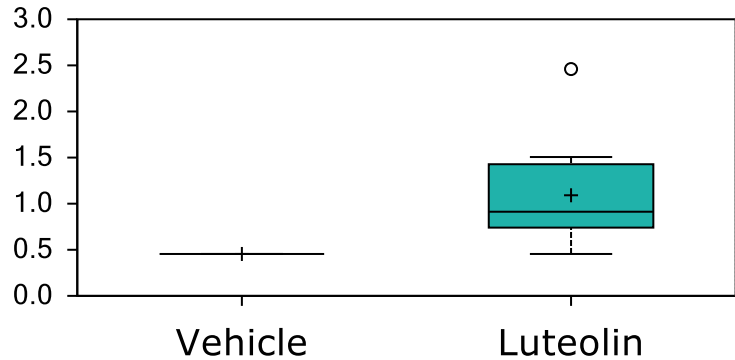

Supplement: Supplementary file 1 [file ijms-22-01321-s001.zip › Figure S2.pdf]
